# A structural constraint for functional interaction between N-terminal and C-terminal domains in simian immunodeficiency virus capsid proteins

**DOI:** 10.1186/1742-4690-7-90

**Published:** 2010-10-18

**Authors:** Natsuko Inagaki, Hiroaki Takeuchi, Masaru Yokoyama, Hironori Sato, Akihide Ryo, Hiroyuki Yamamoto, Miki Kawada, Tetsuro Matano

**Affiliations:** 1 International Research Center for Infectious Diseases, The Institute of Medical Science, The University of Tokyo, 4-6-1 Shirokanedai, Minato-ku, Tokyo 108-8639, Japan; 2 Pathogen Genomic Center, National Institute of Infectious Diseases, 4-7-1 Gakuen, Musashimurayama, Tokyo 208-0011, Japan; 3 Department of Microbiology, Yokohama City University School of Medicine, 3-9 Fuku-ura, Kanazawa-ku, Yokohama 236-0004, Japan

## Abstract

**Background:**

The Gag capsid (CA) is one of the most conserved proteins in highly-diversified human and simian immunodeficiency viruses (HIV and SIV). Understanding the limitations imposed on amino acid sequences in CA could provide valuable information for vaccine immunogen design or anti-HIV drug development. Here, by comparing two pathogenic SIV strains, SIVmac239 and SIVsmE543-3, we found critical amino acid residues for functional interaction between the N-terminal and the C-terminal domains in CA.

**Results:**

We first examined the impact of Gag residue 205, aspartate (Gag205D) in SIVmac239 and glutamate (Gag205E) in SIVsmE543-3, on viral replication; due to this difference, Gag_206-216 _(IINEEAADWDL) epitope-specific cytotoxic T lymphocytes (CTLs) were previously shown to respond to SIVmac239 but not SIVsmE543-3 infection. A mutant SIVmac239, SIVmac239Gag205E, whose Gag205D is replaced with Gag205E showed lower replicative ability. Interestingly, however, SIVmac239Gag205E passaged in macaque T cell culture often resulted in selection of an additional mutation at Gag residue 340, a change from SIVmac239 valine (Gag340V) to SIVsmE543-3 methionine (Gag340M), with recovery of viral fitness. Structural modeling analysis suggested possible intermolecular interaction between the Gag205 residue in the N-terminal domain and Gag340 in the C-terminal in CA hexamers. The Gag205D-to-Gag205E substitution in SIVmac239 resulted in loss of in vitro core stability, which was recovered by additional Gag340V-to-Gag340M substitution. Finally, selection of Gag205E plus Gag340M mutations, but not Gag205E alone was observed in a chronically SIVmac239-infected rhesus macaque eliciting Gag_206-216_-specific CTL responses.

**Conclusions:**

These results present in vitro and in vivo evidence implicating the interaction between Gag residues 205 in CA NTD and 340 in CA CTD in SIV replication. Thus, this study indicates a structural constraint for functional interaction between SIV CA NTD and CTD, providing insight into immunogen design to limit viral escape options.

## Background

One of the characteristics of human immunodeficiency virus (HIV) is to induce persistent viral replication resulting in AIDS progression. HIV has enormous capacity to mutate and escape from host immune recognition, driving genetic diversification of the circulating viruses [1-3]. The Gag capsid (CA), comprising the N-terminal (NTD) and the C-terminal domains (CTD) [4-6], is one of the most conserved proteins in highly-diversified HIVs [7]. Understanding structural constraints in such viral proteins could provide valuable information for immunogen design in AIDS vaccine development.

Virus-specific cytotoxic T-lymphocyte (CTL) responses play a central role in the control of immunodeficiency virus infection [7-12]. CTLs exerting strong suppressive pressure on HIV replication select for viral mutations resulting in escape from CTL recognition [13-16]. Escape mutations in viral proteins with structural constraints are often selected with viral fitness costs, possibly facilitating subsequent immune control [3,17-23]. Thus, conserved viral proteins such as CA can be a promising antigen for vaccine-based CTL induction toward HIV control.

We previously showed vaccine-based control of a simian immunodeficiency virus mac239 (SIVmac239 [24]) challenge in a group of Burmese rhesus macaques possessing the major histocompatibility complex class I (MHC-I) haplotype *90-120-Ia *[19,25]. Gag_206-216 _(IINEEAADWDL) epitope-specific CTL responses play an important role in this control and select for a CTL escape mutation, GagL216S, leading to a leucine (L)-to-serine (S) substitution at the 216th amino acid (aa) in Gag (CA) with the cost of viral fitness [26]. However, *90-120-Ia*-positive vaccinees failed to control a challenge with another pathogenic SIV strain, SIVsmE543-3 [27], that has the same Gag_206-216 _epitope sequence with SIVmac239; Gag_206-216_-specific CTLs did not show responses against SIVsmE543-3 infection due to an aspartate (D)-to-glutamate (E) change, GagD205E, at Gag residue 205 [28].

Thus, the GagD205E substitution in SIVmac239 could result in viral escape from Gag_206-216_-specific CTL recognition. However, in our previous analyses of *90-120-Ia*-positive animals eliciting Gag_206-216_-specific CTL responses for one or two years postchallenge, we observed selection of GagL216S, but not GagD205E mutation in SIVmac239 infection, suggesting a possibility that the GagD205E substitution results in larger reduction of viral replicative ability than GagL216S. In the present study, we first constructed a mutant SIVmac239, SIVmac239Gag205E, with the GagD205E substitution and examined its replication ability in vitro. We found that this amino acid change in the CA NTD results in loss of viral fitness, which can be recovered by an additional amino acid change in the CA CTD. Further analyses presented in vitro and in vivo evidence for a structural constraint in the functional interaction between SIV CA NTD and CTD.

## Results

### Compensation for loss of viral fitness in SIVmac239Gag205E by additional GagV340M substitution

**Figure 1:**
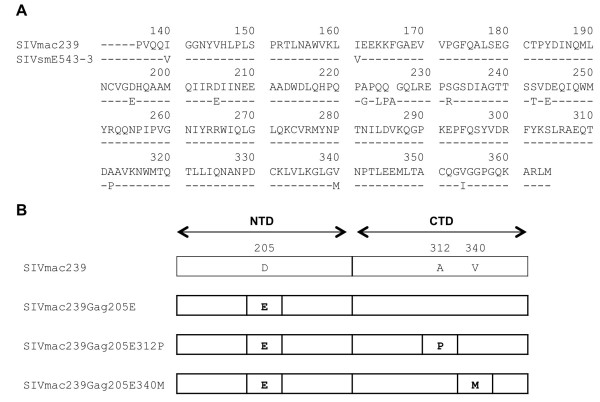
**SIV CA amino acid sequences**. (A) Comparison of SIVmac239 amino acid sequences in CA, Gag residues 136-364, with SIVsmE543-3 (GenBank accession number U72748). (B) Schema indicating the amino acid substitutions in mutant SIV CA.

**Figure 2:**
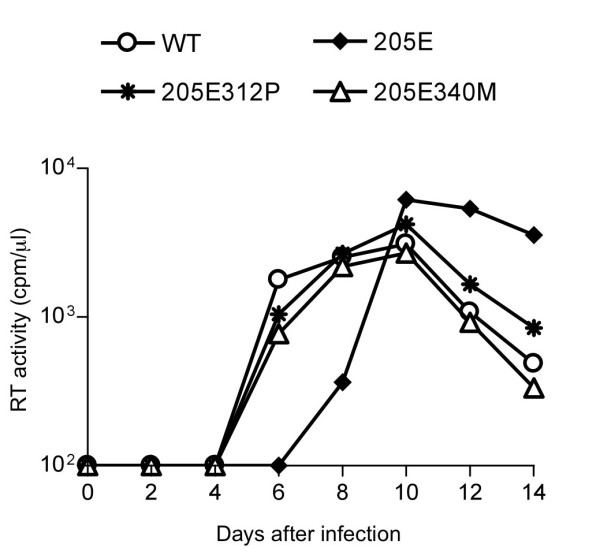
**Wild-type and mutant SIV replication kinetics in HSC-F cells**. HSC-F cells were infected with SIVmac239 (WT, open circles), SIVmac239Gag205E (205E, closed diamonds), SIVmac239Gag205E312P (205E312P, asterisk), or SIVmac239Gag205E340M (205E340M, open triangles). Virus production was monitored by measuring RT activity in the culture supernatants. A representative result from five sets of experiments is shown.

We first constructed a mutant SIVmac239 molecular clone DNA with a mutation of a D-to-E substitution at the 205th aa in Gag (CA NTD) to obtain the mutant virus, SIVmac239Gag205E (Figure 1). Analysis of viral replication kinetics on HSC-F, a macaque T cell line, revealed delayed peak of the mutant SIVmac239Gag205E replication, indicating its lower replicative ability compared to the wild-type SIVmac239 (Figure 2).

**Figure 3:**
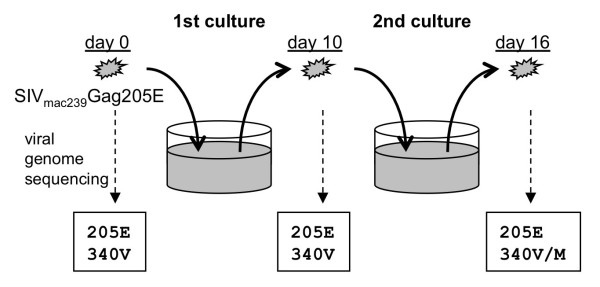
**Passage of SIVmac239Gag205E culture supernatants**. HSC-F cells were infected with SIVmac239Gag205E. The culture supernatant on day 10 was added to fresh HSC-F cells to start the second culture. Viral RNAs were extracted from the first culture supernatant on day 10 and the second culture supernatant on day 16 after the initial infection and subjected to sequence analyses. Dominant amino acid at the 340th residue remained V on day 10 in all cases but was M on day 16 in two of four sets of experiments (Gag340M was detectable on day 10 in these two sets of experiments). No other amino acid change was observed in the CA-coding region.

We further followed up SIVmac239Gag205E replication on HSC-F cells and explored a possibility of viral reversion or additional mutations (Figure 3). No additional gag mutation became dominant on day 10 after SIVmac239Gag205E infection. Interestingly, however, in the second culture after passage of the first culture supernatants on day 10 into uninfected HSC-F cells, an additional mutation, GagV340M, resulting in a valine (V)-to-methionine (M) substitution at the 340th aa in Gag (CA CTD), became dominant in two of four sets of experiments; SIVmac239 has V while SIVsmE543-3 has M at the Gag residue 340. The GagD205E mutation remained dominant, and no other mutations were detected in the CA-coding region even in the second culture.

We then constructed a mutant SIVmac239 molecular clone DNA by introducing the GagV340M mutation into the SIVmac239Gag205E CA-coding region to obtain SIVmac239Gag205E340M (Figure 1). This mutant SIV showed similar replication kinetics on HSC-F cells with the wild-type SIVmac239, indicating compensation for loss of viral fitness in SIVmac239Gag205E by addition of the GagV340M substitution (Figure 2). These results imply that SIV CA with Gag205D-340V or Gag205E-340M combination is functional whereas the CA with Gag205E-340V is less functional.

### Possible interaction between Gag residues 205 and 340 in SIV CA hexamers

Recovery of viral fitness of SIVmac239Gag205E by the GagV340M substitution suggests a possibility of interaction between Gag residues 205 in the NTD and 340 in the CTD. Modeling of CA monomer structure, however, showed that the Gag 205th residue is located in the helix 4 of CA NTD, while the 340th is in the loop between helices 10 and 11 of CTD, which does not support a possibility of intramolecular contact between Gag residues 205 and 340 (data not shown).

**Figure 4:**
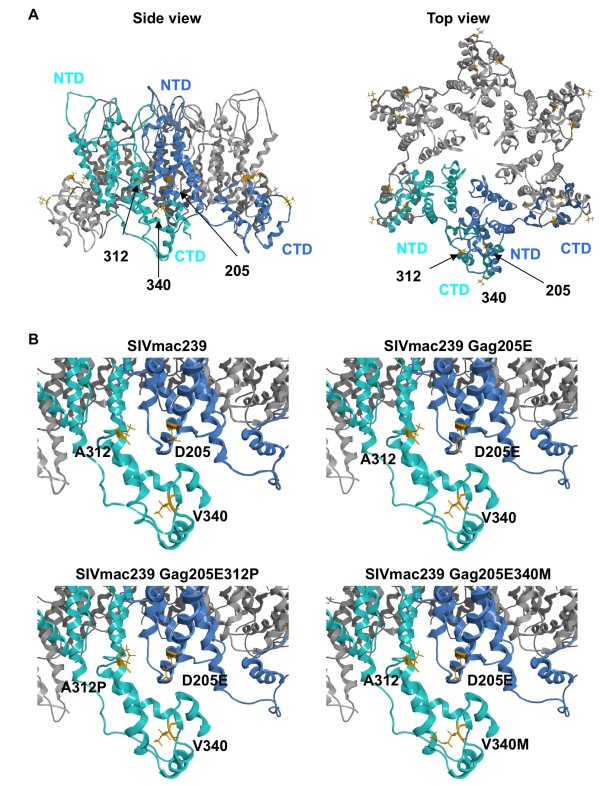
**Structural models of SIVmac239 CA hexamer**. The hexameric SIVmac239 CA models were constructed by homology-modeling using a crystal structure of the hexameric HIV-1 CA at a resolution of 1.90 Å (PDB code: 3H47[33]) as a modeling template. "MOE-Align" and "MOE-Homology" in MOE version 2008.1002 were used for the modeling. The side chains of the 205th, 312th, and 340th aa in Gag are shown as orange sticks. (A) Overall structure of SIVmac239 CA hexamer. (B) The hexameric structures near positions 205, 312, and 340 of wild-type and mutant SIVmac239 CAs.

CA molecules are known to form hexamer lattice in mature virions [29-33]. Modeling of CA hexamer structure revealed that the Gag 205th residue in the NTD is located in close proximity to the 340th in the CTD of the adjacent CA molecule (Figure 4). These observations support a possibility of intermolecular interaction between Gag residues 205 and 340 in CA hexamers.

In addition, the 312th residue in the loop between helices 8 and 9 of CTD is located in close proximity to the 205th in the NTD of the adjacent CA molecule. Because SIVmac239 and SIVsmE543-3 have different amino acids at this residue 312, alanine (A) in the former and proline (P) in the latter, we also constructed a mutant SIVmac239 molecular clone DNA by introducing the GagA312P mutation resulting in A-to-P substitution at the 312th aa in Gag into the SIVmac239Gag205E CA-coding region to obtain SIVmac239Gag205E312P (Figure 1). Analysis of replication kinetics on HSC-F cells indicated recovery of viral fitness by the additional GagA312P substitution in SIVmac239Gag205E (Figure 2).

### Full recovery of viral fitness in SIVmac239Gag205E340M

We then focused on analyzing the possibility of functional interaction between Gag residues 205 in CA NTD and 312/340 in CA CTD. To confirm differences in viral fitness among SIVmac239, SIVmac239Gag205E, SIVmac239Gag205E312P, and SIVmac239Gag205E340M, we compared their replicative ability by viral competition assay (Table 1). The competitions confirmed lower viral fitness of SIVmac239Gag205E compared to wild-type SIVmac239, SIVmac239Gag205E312P, and SIVmac239Gag340M. SIVmac239Gag205E312P showed lower viral fitness than SIVmac239, whereas replication ability of SIVmac239Gag205E340M was no less than the wild-type. These results indicate that the GagD205E substitution in SIVmac239 reduced viral fitness, which was recovered partially by an additional GagA312P and fully by an additional GagV340M substitution. The competition between SIVmac239 and SIVmac239Gag205E340M at the ratio of 1:1 resulted in selection of the latter, suggesting that SIV CA with Gag205E-340M combination observed in SIVsmE543-3 may be slightly more functional than that with Gag205D-340V in SIVmac239.

**Table 1:** Competition between SIV mutants^a^

SIVs in competition	**Ratio of inoc. titers**^**b**^	**Exp. no**.	**Dominant aa sequences**^**c**^			
			
			day 6		day 18	
	4:1	#1	205D		205D	
		#2	205D		205D	
	
SIVmac239 & SIVmac239Gag205E	1:1	#1	205D		205D	
		#2	205D		205D	
	
	1:4	#1	205D		205D	
		#2	205D		205D	

	4:1	#1	205D	312A	205D	312A
		#2	205D	312A	205D	312A
	
SIVmac239 & SIVmac239Gag205E312P	1:1	#1	205D	312A	205D	312A
		#2	205D	312A	205D	312A
	
	1:4	#1	205D	312A	205D	312A
		#2	205D	312A	205D	312A

	4:1	#1	205D	340V	205D	340V
		#2	205D	340V	205D	340V
	
SIVmac239 & SIVmac239Gag205E340M	1:1	#1	205D/E	340V/M	205E	340M
		#2	205D/E	340V/M	205E	340M
	
	1:4	#1	205E	340M	205E	340M
		#2	205E	340M	205E	340M

	4:1	#1	205E	312P	205E	312P
		#2	205E	312P	205E	312P
	
SIVmac239Gag205E & SIVmac239Gag205E312P	1:1	#1	205E	312P	205E	312P
		#2	205E	312P	205E	312P
	
	1:4	#1	205E	312P	205E	312P
		#2	205E	312P	205E	312P

	4:1	#1	205E	340M	205E	340M
		#2	205E	340M	205E	340M
	
SIVmac239Gag205E & SIVmac239Gag205E340M	1:1	#1	205E	340M	205E	340M
		#2	205E	340M	205E	340M
	
	1:4	#1	205E	340M	205E	340M
		#2	205E	340M	205E	340M

^a^HSC-F cells were coinfected with two kinds of SIVs indicated. Viral *gag *fragments were amplified by RT-PCR from viral RNAs from the culture supernatants on days 6 and 18 postinfection and then sequenced. Results from two sets of experiments (Exp. #1 and #2) are shown.

^b^The ratio of the dose (RT activity) of the virus indicated at the top to that at the bottom at coinfection.

^c^Dominant amino acid sequences at the positions where mutations were included in the inoculums are shown. 205D/E, D and E were detected equally at the 205th aa in Gag; 340 V/M, V and M were detected equally at the 340th aa in Gag.

### Inhibition of the early phase of SIVmac239Gag205E replication

**Figure 5:**
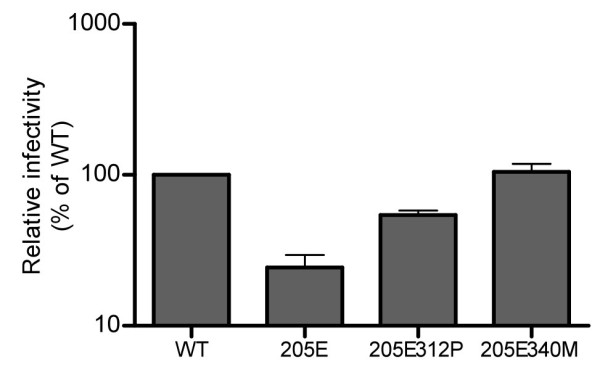
**Mutant SIV infection**. LuSIV cells were infected with SIVmac239 (WT), SIVmac239Gag205E (205E), SIVmac239Gag205E312P (205E312P), or SIVmac239Gag205E340M (205E340M). Luciferase activity was measured 24 hr after infection. Relative infectivity is shown as the ratio (%) of the luciferase activity to that of SIVmac239 (WT). Mean values in three sets of experiments are shown.

**Figure 6:**
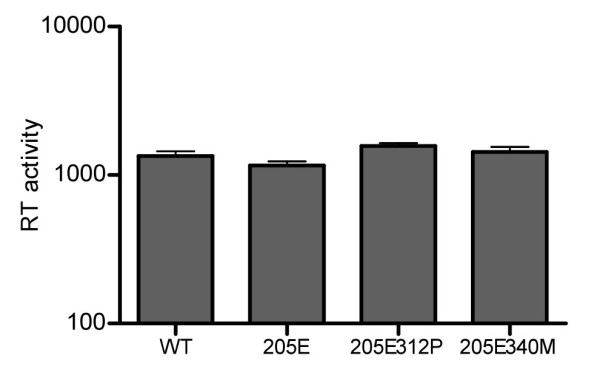
**Mutant SIV production**. COS-1 cells were transfected with molecular clone DNAs of SIVmac239 (WT), SIVmac239Gag205E (205E), SIVmac239Gag205E312P (205E312P), or SIVmac239Gag205E340 M (205E340 M). RT activity of the culture supernatants two days after transfection was measured. Mean values in five sets of experiments are shown.

We examined whether the GagD205E substitution affects the early or late phase of SIVmac239 replication. On LuSIV cells, SIVmac239Gag205E infection showed significantly lower luciferase activity compared to wild-type SIVmac239, SIVmac239Gag205E312P, or SIVmac239Gag205E340M, indicating suppression of the early phase of SIVmac239GagD205E replication (Figure 5). In contrast, we did not find a significant difference in viral production among SIVmac239, SIVmac239Gag205E, SIVmac239Gag205E312P, and SIVmac239Gag205E340M (Figure 6). These results indicate that the loss of viral fitness by the GagD205E substitution is mainly due to inhibition of the early phase of viral replication.

### Loss of in vitro core stability in SIVmac239Gag205E

**Figure 7:**
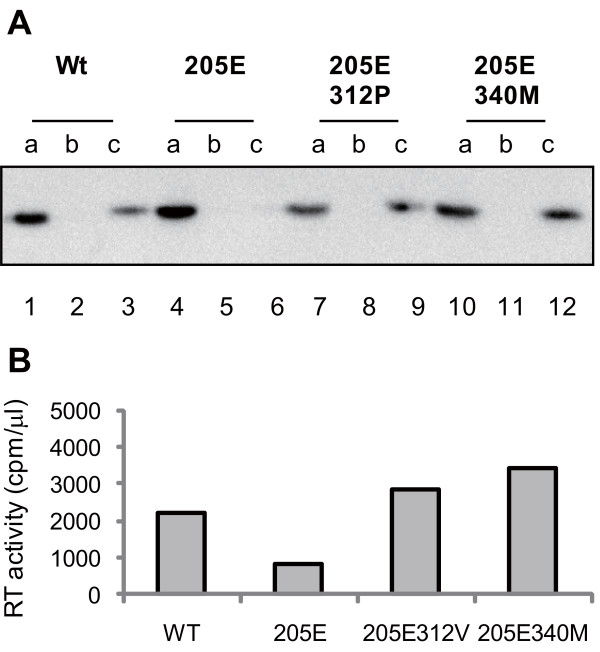
**SIV core stability in vitro**. Concentrated SIVmac239 (Wt; lanes 1-3), SIVmac239Gag205E (205E; lanes 4-6), SIVmac239Gag205E312P (205E312P; lanes 7-9), or SIVmac239Gag205E340M (205E340 M; lanes 10-12) was separated into three fractions (top [a], middle [b], and bottom [c]) by ultracentrifugation under gradient sucrose concentrations in the presence of 0.6% Triton X-100. Each fraction was subjected to Western blot analysis to detect SIV CA p27 proteins (A). A representative result from three sets of experiments is shown. The bottom (c) fractions were also subjected to RT assay (B).

If the GagD205E substitution disturbs intermolecular CA interaction for hexamer formation, it may affect SIV core stability. To assess the core stability in vitro [34], concentrated viruses were separated into three fractions by ultracentrifugation under gradient sucrose concentrations in the presence of Triton X-100 and each fraction was subjected to Western blot analysis to detect CA p27 proteins (Figure 7). In the absence of Triton X-100, CA proteins were detected in the bottom fraction, whereas those in the presence of 1% Triton X-100 were sensitive to the detergent and detected not in the bottom but only in the top fraction (data not shown). We compared the in vitro viral core stability between SIVmac239 and SIVmac239Gag205E in the presence of 0.6%, 0.9%, and 1.35% Triton X-100, respectively, and found a difference in the presence of 0.6% Triton X-100. Additional experiments revealed that SIVmac239Gag205E core was more sensitive to 0.6% Triton X-100 treatment than SIVmac239, SIVmac239Gag205E312P, and SIVmac239Gag205E340M (Figure 7). These results suggest that viral core stability may be reduced by GagD205E substitution but can be recovered by additional GagA312P or GagV340M substitution.

### Selection of GagD205E plus GagV340M mutations in a SIVmac239-infected macaque

The GagD205E substitution results in viral escape from Gag_206-216_-specific CTL recognition. Finally, we examined whether this substitution can be selected in the chronic phase of SIVmac239 infection in *90-120-Ia*-positive macaques eliciting Gag_206-216_-specific CTL responses using plasma samples obtained in our previous experiments [35,36]. SIVmac239-infected *90-120-Ia*-positive macaques select the GagL216S mutation resulting in viral escape from Gag_206-216_-specific CTL recognition, but we found selection of both GagD205E and GagV340M mutations in viral genomes in one animal, R01-007 (Table 2). In this animal, GagD205E and GagV340M mutations were undetectable at week 123 after SIVmac239 challenge, but both became detectable at week 137 and were dominant at week 150. In contrast, the GagL216S mutation dominant at week 123 was not detected at week 150. These results present in vivo evidence indicating functional interaction between the Gag 205th residue in NTD and the 340th in CTD of SIV CA.

**Table 2:** Viral gag sequences in macaque R01-007 infected with SIVmac239^a^

Wks after challenge	**Amino acid sequences**^**b**^		
	
	at 205th	at 216th	at 340th
123	D	S	V
137	D (E)	S (L)	V (M)
150	E	L	M

^a^Viral RNAs were extracted from plasma obtained from a 90-120-Ia-positive macaque R01-007 at weeks 123, 137, and 150 after SIVmac239 challenge. Viral *gag *fragments were amplified by RT-PCR from viral RNAs and then sequenced. This animal showed efficient Gag_206-216_-specific CTL responses and vaccine-based control of a SIVmac239 challenge with rapid selection of the GagL216S escape mutation (at week 5), but accumulated viral mutations in the chronic phase, leading to reappearance of plasma viremia around week 60 after challenge as described previously [19,35].

^b^Dominant amino acid sequences at the 205th, 216th, and 340th aa in Gag are shown. Parentheses indicate the sequences that are not dominant but detectable.

## Discussion

The Gag CA which is one of the most conserved proteins in HIV and SIV may be a promising immunogen for CTL-based AIDS vaccines. However, the limitations imposed on amino acid sequences in CA are not fully understood. In the present study, we found that the GagD205E substitution in SIVmac239 CA NTD reduces viral fitness, which is recovered by additional GagA312P or GagV340M substitution in the CTD. SIVmac239Gag205E passaged in cell culture often resulted in selection of an additional GagV340M mutation. Furthermore, selection of Gag205E plus Gag340M mutations, but not Gag205E alone, was observed in a chronically SIVmac239-infected rhesus macaques. These results provide evidence indicating a functional interaction between Gag residues 205 in CA NTD and 340 in CA CTD, presenting a structural constraint for functional interaction between SIV CA NTD and CTD.

HIV and SIV Gag proteins are expressed as unprocessed polyproteins, which are assembled and incorporated into the virions. Concomitant with viral budding, incorporated Gag polyproteins are proteolytically cleaved by viral protease into processed proteins including MA (matrix), CA, and NC (nucleocapsid), participating in mature infectious virion formation [37,38]. Recent structural analyses [31-33,39-41] indicated that CA proteins form hexamer lattice in matured virions; in the mature CA core, the intermolecular NTD-NTD and NTD-CTD interfaces are involved in the formation of CA hexamers, while the intermolecular CTD-CTD interface connects neighboring hexamers. Our modeling analyses did not support a possibility of intramolecular interaction but indicated possible intermolecular interaction between Gag205 in CA NTD and Gag312/340 in CA CTD, which may affect CA hexamer formation during viral maturation. This is consistent with our results in Figure 5 indicating that the GagD205E substitution results in inhibition of the early phase of SIVmac239 replication, which can be overcome by additional GagA312P or GagV340M substitution. This possibility is supported also by our results on viral core stability in vitro, although it remains unclear how much extent the core stability in vitro can reflect the one in vivo [42]. There has been no report suggesting the influence of the Gag 205 residue on SIV sensitivity to tripartite interaction motif 5α (TRIM5α). A previous report on HIV CA lattice [31,43] indicated a potential interaction between the helix 4 of NTD and the loop connecting helices 10 and 11 of CTD in the adjacent molecule. Our results suggest the possible involvement of Gag205 and Gag340 residues in this intermolecular NTD-CTD interaction in CA hexamers.

The molecular model of CA hexamers incorporating the GagD205E substitution suggested shortening of the distance between Gag205 and Gag340 residues, which looked to be compensated by GagV340M substitution (Figure 4). The modeling can draw a hydrophobic pocket between Gag205 and Gag340 residues in SIVmac239Gag205E340M as well as SIVmac239, but not in SIVmac239Gag205E CA hexamers. Thus, this pocket may be a target candidate for anti-viral drugs.

Both GagL216S and GagD205E mutations can result in escape from Gag_206-216_-specific CTL recognition [19,28], but the former is usually selected in SIVmac239-infected *90-120-Ia*-positive macaques probably because the latter reduces viral fitness more severely than the former. In this study, we found selection of GagD205E plus GagV340M mutations in the chronic phase of SIVmac239 infection in a *90-120-Ia*-positive macaque. In this animal, the CTL escape GagL216S mutation first selected after SIVmac239 challenge became undetectable and was replaced with the CTL escape GagD205E mutation in combination with GagV340M in the chronic phase. This may imply that the GagD205E plus GagV340M mutations might be more advantageous than the GagL216S mutation for SIVmac239 replication in the presence of Gag_206-216_-specific CTL pressure.

We observed the addition of GagV340M mutation but not a Gag205E-to-Gag205D reversion in SIVmac239Gag205E passage. This may be due to difference in frequencies between purine-to-purine (guanine-to-adenine) change in the former and purine-to-pyrimidine (adenine-to-thymine) change in the latter. The appearance of additional GagV340M mutation in SIVmac239Gag205E passaged in cell culture as well as the selection of GagD205E plus GagV340M mutations in an animal provides key evidence indicating functional interaction between Gag residues 205 in CA NTD and 340 in CA CTD. The Gag is a promising candidate as a vaccine immunogen for CTL induction, because cumulative studies have indicated the efficacy of Gag-specific CTL responses against HIV and SIV infection [7,25,44,45]. However, viral mutational escape from CTL recognition is a major challenge for AIDS vaccine design. Thus, the information on the structural constraint presented in this study might be helpful for immunogen design in AIDS vaccine development.

## Conclusions

Our results present in vitro and in vivo evidence implicating the interaction between Gag residues 205 in CA NTD and 340 in CA CTD in SIV replication. SIV CA with Gag205D-340V (observed in SIVmac239) or Gag205E-340M combination (observed in SIVsmE543-3) is functional whereas the CA with Gag205E-340V is less functional. Thus, the present study indicates a structural constraint for functional interaction between SIV CA NTD and CTD, providing valuable information for immunogen design to limit viral escape options.

## Methods

### Analysis of mutant SIV replication

SIV molecular clone DNAs with gag mutations were constructed by site-directed mutagenesis from the wild-type SIVmac239 molecular clone DNA [24]. Virus stocks were obtained by transfection of COS-1 cells with wild-type or mutant SIV molecular clone DNAs using Lipofectamine LTX PLUS (Invitrogen, Tokyo, Japan). Viral titers were measured by reverse transcription (RT) assay as described previously [46]. For analysis of viral replication kinetics, HSC-F cells (herpesvirus saimiri-immortalized macaque T-cell line) [47] were infected with wild-type or mutant SIVs (normalized by RT activity), and virus production was monitored by measuring RT activity in the culture supernatants. To examine viral infectivity, LuSIV cells, which are derived from CEMx174 cells and contain a luciferase indicator gene under the control of the SIVmac239 long terminal repeat, were cultured for 24 hr after viral infection and then lysed in a reporter lysis buffer (Promega Corp., Tokyo, Japan) for measurement of the luciferase activity in a luminometer (GloMax™ 96 Microplate Luminometer, Promega Corp.).

### Viral competition assay

HSC-F cells were coinfected with two SIVs at a ratio of 1:1 or 1:4, and the culture supernatants harvested every other day were used for RT assays. On day 6, the supernatant was added to fresh HSC-F cells to start the second culture. Similarly, on day 12 after the initial coinfection, the second culture supernatant was added to fresh HSC-F cells to start the third culture. RNAs were extracted using the High Pure viral RNA kit (Roche Diagnostics, Tokyo, Japan) from the initial culture supernatant on day 6 and from the third culture supernatant on day 18 post-coinfection. The fragment (nucleotides 1231 to 2958 in SIVmac239 [GenBank accession number M33262]) containing the entire gag region was amplified from the RNA by RT-PCR and sequenced to determine dominant sequences as described previously [19].

### Molecular modeling of hexameric SIVmac239 CA

The crystal structures of HIV-1 CA NTD at a resolution of 2.00 Å (PDB code: 1M9C[48]), HIV-1 CA CTD at a resolution of 1.70 Å (PDB code: 1A8O[5]), and hexameric HIV-1 CA at a resolution of 1.90 Å (PDB code: 3H47[33]) were taken from the RCSB Protein Data Bank [49]. Three-dimensional (3-D) models of monomeric SIVmac239 CA were constructed by the homology modeling technique using 'MOE-Align' and 'MOE-Homology' in the Molecular Operating Environment (MOE) version 2008.1002 (Chemical Computing Group Inc., Quebec, Canada) as described [50,51]. We obtained 25 intermediate models per one homology modeling in MOE, and selected the 3-D models which were the intermediate models with best scores according to the generalized Born/volume integral methodology [52]. The final 3-D models were thermodynamically optimized by energy minimization using an AMBER99 force field [53] combined with the generalized Born model of aqueous solvation implemented in MOE [54]. Physically unacceptable local structures of the optimized 3-D models were further refined on the basis of evaluation by the Ramachandran plot using MOE. The structures of hexameric SIVmac239 CA were generated from the monomeric structures by MOE on the basis of the assembly information of hexameric HIV-1 CA crystal structure [33].

### Analysis of viral CA core stability in vitro

Detergent treatment of wild-type and mutant SIV particles was performed essentially as described previously [34]. Briefly, viruses from COS-1 cells transfected with viral molecular clone DNAs (normalized by RT activity) were concentrated by ultracentrifugation at 35,000 × rpm for 75 min at 4°C in a SW41 rotor (Beckman Instruments, Tokyo, Japan) through a cushion of 20% sucrose in phosphate buffered saline (PBS). The concentrated viral pellets were suspended in PBS. Sucrose step gradients were prepared in SW55 centrifuge tubes with the 2.0 ml layer of 60% sucrose on the bottom and 2.1 ml layer of 20% sucrose overlaid. Then, 0.1 ml of Triton X-100 in PBS and 0.5 ml of concentrated viruses were overlaid and ultracentrifuged at 35,000 × rpm for 60 min at 4°C in a SW55Ti rotor (Beckman Instruments). Three fractions (top [a], middle [b], and bottom [c]) of 1.1 ml each were collected from the top and subjected to Western blot analysis using plasma from a simian-human immunodeficiency virus 89.6PD-infected rhesus macaque [55] and RT assay.

## Competing interests

The authors declare that they have no competing interests.

## Authors' contributions

NI and TM designed the study. NI, HT, and AR performed virological analyses in vitro. MY and HS performed structure modeling analyses. HY and MK examined viral genome sequences. NI and TM analyzed the data and wrote the paper. All authors read and approved the final manuscript.
